# The Route to an Integrative Associative Memory Is Influenced by Emotion

**DOI:** 10.1371/journal.pone.0082372

**Published:** 2014-01-10

**Authors:** Brendan D. Murray, Elizabeth A. Kensinger

**Affiliations:** Department of Psychology, Boston College, Chestnut Hill, Massachusetts, United States of America; University of Texas at Dallas, United States of America

## Abstract

Though the hippocampus typically has been implicated in processes related to associative binding, special types of associations – such as those created by integrative mental imagery – may be supported by processes implemented in other medial temporal-lobe or sensory processing regions. Here, we investigated what neural mechanisms underlie the formation and subsequent retrieval of integrated mental images, and whether those mechanisms differ based on the emotionality of the integration (i.e., whether it contains an emotional item or not). Participants viewed pairs of words while undergoing a functional MRI scan. They were instructed to imagine the two items separately from one another (“non-integrative” study) or as a single, integrated mental image (“integrative” study). They provided ratings of how successful they were at generating vivid images that fit the instructions. They were then given a surprise associative recognition test, also while undergoing an fMRI scan. The cuneus showed parametric correspondence to increasing imagery success selectively during encoding and retrieval of emotional integrations, while the parahippocampal gyri and prefrontal cortices showed parametric correspondence during the encoding and retrieval of non-emotional integrations. Connectivity analysis revealed that selectively during negative integration, left amygdala activity was negatively correlated with frontal and hippocampal activity. These data indicate that individuals utilize two different neural routes for forming and retrieving integrations depending on their emotional content, and they suggest a potentially disruptive role for the amygdala on frontal and medial-temporal regions during negative integration.

## Introduction

A critical feature of human memory is the ability to form novel associations between pieces of information. Some special types of associations, such as integrations via interactive imagery [1]–[2], involve binding those discrete pieces of information into a single mental representation (e.g., a “shirt” and the color “blue” are remembered as a “blue shirt” or the combination of “poached egg, ham, and cheese” is remembered as a “croque madame”). These types of integrated representations are useful because their retrieval from memory typically allows access to multiple related features or items at once. When “blue shirt” is retrieved, a person can simultaneously bring to mind the color and the shape of the object in question, and when “croque madame” is listed on a menu, it signifies to the customer the particular set of ingredients it contains.

Although processes related to associative binding have typically been ascribed to the hippocampus (“HC”) [3]–[4], patients with damage to the HC show a mnemonic advantage for remembering unitized representations [5]–[6]. These findings suggest that memory for integrations may not rely on the hippocampus. Neuroimaging studies have emphasized a role for visual processing regions or regions of the parahippocampal gyrus (“PHG”) [7]–[10].

These previous investigations have used neutral stimuli. However, we generate many integrations that contain both emotional and non-emotional content. For example, with the recent bombings at the 2013 Boston Marathon, most people formed an association between the previously unrelated concepts of “bomb” and “marathon,” such that “marathon bomb” now brings to mind that particular event. We have previously shown that integrations including an emotional item are created faster than integrations containing only neutral items, though those integrations are also less likely to be maintained in memory [2]. These behavioral results suggest a dissociation between the processes leading to integration with emotional items and with neutral items, but the basis for this dissociation is unknown. When engaged by emotional events, amygdala activity often modulates the processing within sensory regions [11]–[14] and also processing within the HC [15]–[16] and other MTL regions [17]–[18]. Emotion can also influence the allocation of elaborative processing resources [19]–[20]. It is, therefore, possible that the facilitated integration could stem from emotion's modulation of binding processes (e.g., within the MTL), of perceptual processes (e.g., within visual regions), or of conceptual processes (e.g., within prefrontal regions) [12], [21] for a discussion of how these different processes may affect integration]. Based on our prior research, demonstrating that integration with emotional items can be achieved faster than integration with neutral items – but that such integrations are also less likely to be retained in memory over long-term delays [2] – we hypothesized that emotion would enhance the connection between visual activity and integrative ability but might reduce the reliance on MTL or prefrontal processes for the integration process.

The present study is the first to examine the effects of negative emotion on the neural processes recruited during the formation and retrieval of integrated representations. Participants performed an integrative and a non-integrative mental imagery task followed by an associative recognition task, all while undergoing an fMRI scan. We show that emotion affects the processes used to form and retrieve vivid integrations: Imagining neutral integrations selectively recruited activity in lateral PFC and right posterior PHG, and this activity increased parametrically with self-reported integration success; conversely, imagining negative integrations selectively recruited activity in cuneus, and this activity increased parametrically with self-reported integration success. Connectivity analyses suggested that amygdala engagement during the creation of integrations might result in a disengagement of HC and PFC processes, providing further evidence that emotion affects the neural processes supporting integration.

## Methods

### Ethics Statement

This study was conducted in accordance with to the principles of Declaration of Helsinki. Procedures were approved by the Institutional Review Boards of Boston College and Massachusetts General Hospital.

### Participants

Participants were 20 right-handed young adults (11 female) aged 18–30 (*M* = 24.4). Three participants (2 female) were excluded for excessive motion (greater than 5 degrees of translation or rotation in any of the three movement axes) for a final sample size of 17. Participants provided informed written consent in a manner approved by the Boston College and Massachusetts

General Hospital Institutional Review Boards (see above), and were remunerated at $25 per hour. For each participant, anatomical MR data were collected prior to the encoding phase of the study, and functional MR data were collected during both the encoding and recognition phases.

### Stimuli

Stimuli were 300 semantically-unrelated verbal pairs generated pseudorandomly from negative and neutral words in the Affective Norms for English Words [22] and Kucera & Francis [23] series. Following randomization, pairs were hand-checked to ensure no pre-existing semantic relationship between the two referents. Negative words had a mean valence rating of 2.7 (on a scale from 1 = “extremely unpleasant” to 9 = “extremely pleasant”) and a mean arousal rating of 5.7 (1 = “extremely calming” to 9 = “extremely agitating”), and neutral words were matched to the negative words on frequency, word length, and imageability. 240 pairs were presented during encoding, with the remaining 60 pairs serving as new “lures” during recognition. At recognition, 120 of the studied pairs were presented as intact and 120 were presented as rearranged. At encoding and retrieval, half of the presented pairs in each condition – non-integrative and integrative imagery at encoding; intact, rearranged, and new pairs at recognition – contained a negative word paired with a neutral word (“negative” pairs) and half contained two neutral words (“neutral” pairs). Studied pairs at encoding and new pairs that appeared at retrieval were varied across participants.

### MRI Anatomical Scan

All imaging was performed on a 1.5T Siemens Avanto full-body magnetic resonance scanner (Erlangen, Germany) with all images acquired using a high-resolution 32-channel head coil. Anatomical images were collected using a multiplanar rapidly-acquired gradient echo (MP- RAGE) sequence in an sagittal orientation, with field of view = 256×256, matrix = 256×256, and slice thickness of 1.33 mm (no gap) to yield 1×1×1.33 mm voxel resolution (TR/TE = 2730/3.31 ms, flip angle = 40°).

### MRI Functional Scan

#### Image acquisition

BOLD-weighted functional images were obtained using a T2*- weighted echoplanar imaging sequence, with 26 interleaved 3.12 mm (0.6 mm skip) slices oriented parallel to the long axis of the hippocampus acquired within each volume (TR/TE = 2000/40 ms, field of view = 256 mm, 64×64 mm acquisition matrix, flip angle = 90°). A total of 296 volumes were acquired during each of four encoding runs, and 196 volumes were acquired during each of four recognition runs. Where full-brain coverage was not available because of slice orientation, we ensured full coverage of the ventral temporal lobe and PFC for each participant.

#### Procedure

Encoding was divided into four runs. In the first two runs participants were instructed to perform non-integrative mental imagery and in the latter two runs were instructed to perform integrative mental imagery. As described in Murray and Kensinger [2], imagery order was not counterbalanced because previous testing indicated participants were unable to successfully perform non-integrative imagery after learning of integrative imagery. During each of the two non-integrative runs, participants viewed 60 word pairs (30 negative pairs and 30 neutral pairs, randomly intermixed) for five seconds each and were instructed to generate separate mental images for each item in as much vivid detail as possible. They were explicitly instructed not to imagine the items together or interacting in any way. After five seconds, the pair disappeared from the screen and participants were given two seconds to rate their imagery success on a scale from 1 (“no image for one or both items” or “I could only imagine the items together”) to 4 (“I could imagine each item, separately, in very vivid detail”). Pairs that the participant failed to rate within two seconds (fewer than 1% of all trials) were automatically given a rating of “1”. During each of the two integrative runs, participants viewed 60 different word pairs and were instructed to generate a mental image that combined both items into a single mental representation. Pairs were again shown for five seconds, followed by the two-second rating period (1 = “no image generated” or “I could only imagine the items separately”; 4 = “extremely vivid image that incorporates both items”) and one second of fixation. Trials were jittered with a variable duration sensorimotor baseline task [24] in which arrows that randomly pointed to the left or right were presented for one second, and participants had to make a button press corresponding to the direction in which the arrow was pointing. The arrow task lasted for the duration of the inter-trial interval (2–12 s).

We chose a 5-second encoding trial because our behavioral research [2] revealed that with this length of trial, behavioral effects of emotion on memory are minimized. We reasoned that, if performance differed between the emotional and neutral pairs, this could create difficulty with the interpretation of neural differences. First, there would be a different number of trials that were remembered in the emotional and neutral conditions, and different numbers of trials encoded at each level of success used for the parametric analyses. These differences could confound the ability to isolate effects due to emotion. Second, if neural processes differ when behavioral performance between the emotional and neutral pairs are matched, this makes it more likely that the neural differences are related to the emotional content of the pairs and not to other features of the memory (confidence, strength, etc) that may differ.

Following the encoding runs, participants were removed from the scanner, given instructions and practice trials for a surprise recognition task, and then returned to the scanner. After a brief localizer scan, participants performed the recognition task. The recognition test took place approximately 30 minutes after encoding. The recognition task was divided into four runs, each consisting of 30 pairs that were intact from study, 30 pairs that were rearranged from study, and 15 pairs that were new “lures”. Each pair appeared on the screen for four seconds, during which time participants made a button press to indicate whether the pair was intact, rearranged, or new. Test pairs were randomly jittered with the sensorimotor baseline task.

#### Imaging data preparation and analysis

Functional images were temporally and spatially preprocessed using the Statistical Parametric Mapping (SPM5) software (Wellcome Trust Centre for Neuroimaging). Slices in each volume were temporally synchronized to the first slice acquired, realigned using six motion parameters to correct for movement, and spatially normalized to the Montreal Neurological Institute anatomical template. Images were smoothed using a 7.6 mm isotropic full-width half- maximum Gaussian kernel. Data were concatenated across the four runs (separately for the encoding and recognition phases) and regressors were added to all first-level analyses to correct for linear drift in the MR signal. Imaging data were prepared separately for the encoding runs and recognition runs, but the parameters were the same for both memory phases.

Statistical analyses were performed using the SPM5 general linear model (GLM). Event- related analyses were conducted. At encoding, separate regressors were entered for each combination of encoding strategy (non-integrative; integrative), emotionality (negative; neutral) and subsequent memory (hits, defined as correct subsequent identification of intact pairs *or* correct subsequent identification of both recombined pairs containing the referents of the encoded pair [25]; misses). Retrieval trials were divided by encoding strategy, emotionality, and accuracy (hits, defined by correct identification of an intact *or* recombined pair; misses). Parametric modulation regressors were modeled for each condition, representing participants' imagery success ratings (i.e., their 1–4 imagery ratings); all participants provided a sufficiently large number of each of the four ratings for this to be possible. At retrieval, if the items in a rearranged pair came from two encoding pairs that received different ratings, the lower rating was used as the parametric regressor value). Also modeled were nuisance regressors modeling linear drift in the MR signal for each run, and for retrieval runs we modeled a separate regressor representing all “new” trials. Separately for encoding and retrieval, the eight conditions of interest were submitted to a repeated-measures ANOVA with encoding strategy, emotionality, and subsequent memory (encoding) or accuracy (retrieval) as factors. The four parametric modulation contrasts associated with correct memory also were submitted to a repeated-measures ANOVA with encoding strategy and emotionality as factors.

To validate the location of the brain regions reported here, each active cluster was masked using the Neuroinformatics Volume of Interest tool [26], which maps volumes of interest from the BrainMap database [27]. Any named region that we identify here in the reporting of results (e.g., “parahippocampal gyrus”) is overlapped by the corresponding region in the database.

#### Psychophysiological interaction (PPI) analysis

**Figure 1:**
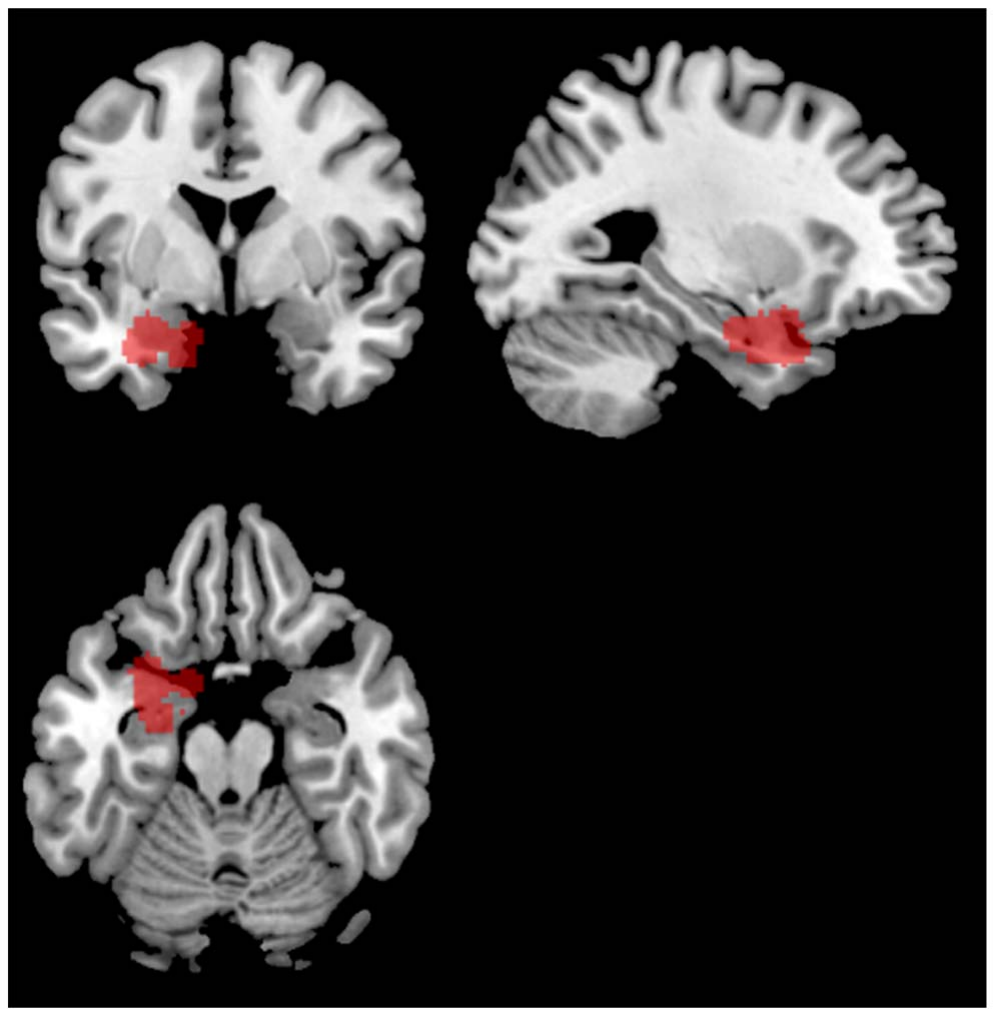
Composite map of all participants' volumes of interest used for PPI analysis.

All PPI analyses were conducted using the PPI toolbox built in to the SPM5 software. Volumes of interest (VOI) for the left amygdala seed region were defined functionally at the single-subject level by contrasting negative integration>neutral integration, and drawing a 6 mm VOI around the peak voxel in left amygdala for each participant (Fig. 1; see Table 1 for individual peak coordinates). For each subject, a PPI was run for the interaction between negative integration and neutral integration, weighting those conditions +1 and −1 respectively while weighting all other conditions 0. The contrast images generated from these PPIs were then submitted to a second-level one-sample *t*- test to show which regions were more positively – or less negatively – correlated with left amygdala for negative integration than neutral integration, and then submitted to a one-sample *t*- test to show which regions were more positively – or less negatively – correlated with left amygdala for neutral integration than negative integration.

**Table 1:** Peak voxel coordinates (MNI) of left amygdala VOIs for each participant.

Participant	x	y	z
1	−32	12	−28
2	−28	8	−28
3	−16	−2	−28
4	−16	−6	−26
5	−30	0	−28
6	−32	4	−28
7	−32	6	−28
8	−16	0	−20
9	−20	−6	−30
10	−16	2	−30
11	−28	0	−22
12	−34	8	−26
13	−24	4	−24
14	−26	−6	−24
15	−14	4	−24
16	−24	−8	−22
17	−28	8	−20

To isolate the directionality of the correlations for negative and neutral integration, separate PPI analyses were run at the single-subject level for negative integration>baseline (i.e., weighting the negative integrative condition +1 and all other conditions 0) and for neutral integration>baseline. The same VOIs defined for the full interaction were used to define the seed regions for both of these analyses. The contrast images generated from these PPI analyses were then submitted to separate second-level one-sample *t*-tests to reveal left amygdala correlations with other brain regions during negative integration and neutral integration. To determine directionality, we inclusively masked these second-level baseline contrasts with the relevant contrast from the second-level interaction (i.e., negative>baseline and baseline>neutral were each masked with negative>neutral, and neutral>baseline and baseline>negative were each masked with neutral>negative). In this way, the results of the baseline contrasts were constrained to only show activity in regions that were also active in the full interaction. For example, if a region reached threshold in the negative>neutral and negative>baseline contrasts but not the baseline>neutral contrast, then logically that region must be positively correlated with left amygdala for negative integration. These combined masks were thresholded at *P*<0.01, unless otherwise noted.

#### Correction for multiple comparisons

We ran a Monte Carlo simulation [28] to determine the cluster extent threshold necessary to yield a corrected *P*<0.05. This simulation indicated that a cluster extent of 8 voxels or greater, at an uncorrected threshold of *P*<0.001, would have a corrected Type 1 error rate of *P*<0.05. All reported analyses use this threshold, except where otherwise noted (i.e., regions for which we had an *a priori* hypothesis, such as visual regions during negative integration).

## Results

### Behavioral Results

Corrected recognition data (hits [saying “recombined” to a recombined pair or “intact” to an intact pair] minus false alarms [saying “intact” or “recombined” to a new pair]) for all successfully-imagined pairs (rated a “3” or “4” at encoding) were submitted to a 2 (encoding strategy: non-integrative, integrative) ×2 (emotionality: negative, neutral) repeated-measures ANOVA, which revealed that pairs studied integratively (*M*_negative_ = 56.5%, *SE* = 3.7%; *M*_neutral_ = 55.8%, *SE* = 3.6%) were better recognized than those studied non-integratively (*M*_negative_ = 43.4%, *SE* = 2.2%; *M*_neutral_ = 44.8%, *SE* = 2.5%; *F*(1,16) = 24.25, *P*<0.001). No effect of emotion was observed, nor did emotion and encoding strategy interact. The absence of an emotion effect was unsurprising, as we specifically selected an encoding presentation time – five seconds – that had not shown previous behavioral effects on recognition memory [2]. In this way, we could ensure that any neural effects of emotion would not be confounded with differences in encoding or retrieval success for emotional or neutral pairs.

### Neuroimaging results

#### Parametric effects of integrative imagery success that precedes correct recognition

**Figure 2:**
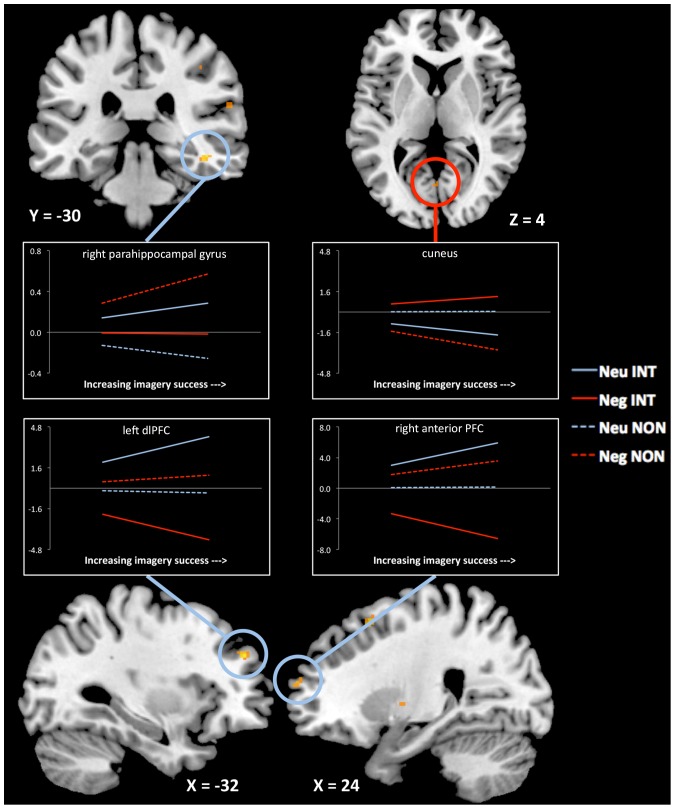
Regions that show significant parametric increase during encoding as a function of successful integration. Right PHG (44 −30 −16), left dorsolateral PFC (−32 46 30), and right anterior PFC (24 62 16) all show selective parametric increase during neutral integration. The reversed interaction reveals that cuneus (0 −66 4) shows selective parametric increase during negative integration. Lines represent beta weights (slope) for the parametric relation, extracted from the peak voxel within each cluster. Dashed lines represent weights from the non-integrative condition and solid lines represent weights from the integrative condition. † = full interaction is significant at *P*_uncorrected_<0.005; * = full interaction is significant at *P*_corrected_<0.05; ** = pairwise comparison (e.g., neutral integration>negative integration) is significant at *P*_corrected_<0.05.

We used parametric modulation analysis to reveal which brain regions showed a linear increase in activity as a function of participants' ratings of mental imagery vividness and success. We restricted the analysis to pairs that were successfully recognized, to ensure that the activity revealed by the analysis was reflective of the integration process and not of other memory demands. We examined the regions that showed a significant interaction between emotion and encoding strategy in a second-level 2×2 ANOVA. Regions including the right posterior PHG, left dorsolateral PFC and right anterior PFC showed disproportionate increases in activity during the integration of neutral pairs (see Fig. 2; see Table 2 for all peak activations and voxel extents).

**Table 2:** Regions showing significant parametric correspondence to vividness ratings during encoding of neutral integrations.

Region	Laterality	x	y	z	*t*	*k*
DLPFC (BA9)	L	−32	46	30	3.88	14
Posterior HC/PHG (BA36)	R	44	−30	−12	3.86	9
Precentral gyrus (BA6)	L	−54	4	8	3.84	44
Primary motor area (BA6)	R	24	16	54	3.84	28
Anterior HC (BA34)	L	−12	−2	−22	3.77	16
Posterior insula (BA13)	R	50	−10	6	3.72	10
Anterior PFC (BA10)	R	24	62	16	3.47	8

Reversing the interaction contrast, no regions were found to track selectively with increasing imagery success for negative integration at the corrected level. However, dropping the threshold to *P*_uncorrected_<0.005 revealed a region of cuneus (BA 17/18; 0, −66, 4, *k* = 5) that increased selectively for integration of negative pairs (Fig. 2).

Confirming the dissociation between regions that tracked parametrically with successful integration of neutral items and of negative items, a conjunction analysis revealed no regions that showed a stronger parametric correspondence to both neutral integrative vs. non-integrative success and also negative integrative vs. non-integrative success, even when the threshold was dropped to *P*_uncorrected_<0.005.

#### Parametric effects of prior imagery success during successful recognition

**Figure 3:**
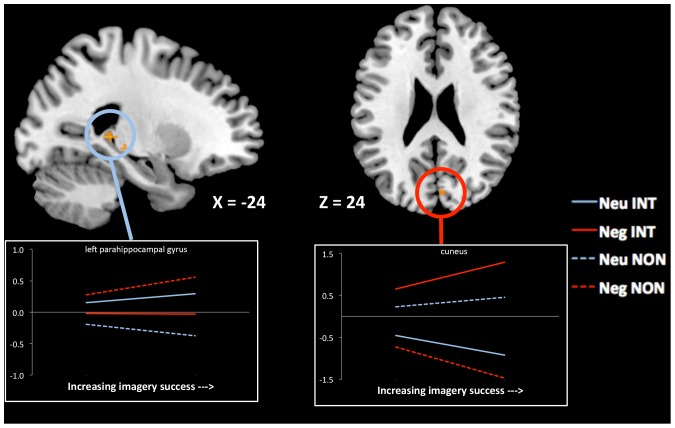
Regions that show significant parametric increase during retrieval as a function of prior successful visual integration. Left PHG (−24, −36, 4,) shows selective parametric increase during neutral integration. Cuneus (6 −80 24) shows selective parametric increase during negative integration. Lines represent beta weights for the parametric relation, extracted from the peak voxel within each cluster. Dashed lines represent weights from the non-integrative condition and solid lines represent weights from the integrative condition. † = full interaction is significant at *P*_uncorrected_<0.005; * = full interaction is significant at *P*_corrected_<0.05.

We again used parametric modulation analysis to determine whether imagery vividness/success ratings would influence neural activity during successful retrieval differently for emotional and neutral pairs. In other words, how does the vividness of the integration created during encoding influence the activity corresponding to its successful retrieval? A second-level 2×2 ANOVA (encoding x emotion, for hits only) revealed a region of left posterior PHG (−24, −36, 4, *k* = 10) whose parametric correspondence was strongest for neutral pairs that were studied integratively (Fig. 3). Though no regions showed the strongest parametric correspondence for negative integration at the corrected threshold, dropping the threshold to *P*_uncorrected_ = 0.005 revealed a region of cuneus (6, −80, 24, *k* = 5), and when a small volume correction was used, this cuneus activity reached the corrected significance level of P<.05. A conjunction analysis revealed that only a region of left angular gyrus (−60 −52 32, *k* = 15) showed stronger parametric correspondence to both neutral integrative vs. non-integrative and negative integrative versus non-integrative retrieval.

#### Non-parametric effects of emotion and encoding strategy on subsequent memory

Though the above analyses focused on the successfully remembered pairs, we also were interested in examining how emotion would affect the processes leading to successful (vs. unsuccessful) memory for integrated pairs. For this analysis, parametric modulation analyses are less informative (because, among other reasons, the ratings of imagery success become largely unbalanced across conditions when analyzing subsequent misses). Instead, we conducted a standard, random-effects whole-brain analysis. In the full interaction (encoding x emotionality x subsequent memory), no regions selectively related to subsequent hits for neutral pairs studied integratively. However, a region of precuneus (BA 31; −16, −52, 24, *P*<0.001, *k* = 5) selectively corresponded with subsequent memory during the integrative encoding of negative pairs, and dropping the *P* threshold to 0.005 revealed a region of left amygdala (−10 6 −12, *k* = 9) as well. When using a small volume correction, this amygdala activity reached a corrected significance level of P<.05.

As further evidence that the precuneus and amydala were selectively active during the integrative encoding of negative but not neutral pairs, when we directly contrasted, for neutral pairs, integrative encoding trials that preceded subsequent correct memory to non-integrative encoding trials that preceded subsequent memory, this contrast revealed only a region of left PHG (BA 36; −30 −54 −2, *k* = 20), with no voxels within visual processing regions or amygdala revealed, even if the significance threshold were lowered to *P*_uncorrected_<0.005.

#### Non-parametric effects of emotion and encoding strategy on recognition performance

For the full interaction (encoding strategy x emotion x accuracy), no voxels survived, even at *P*_uncorrected_<0.005. Similarly, no voxels survived any of the two-way interactions (emotion x accuracy, encoding strategy x accuracy, or emotion x encoding strategy).

#### Connectivity analysis: Psychophysiological interactions (PPI) between left amygdala and other brain regions during negative and neutral integration

Because we had an *a priori* hypothesis that effects of emotion may be related to amygdala activity, we conducted PPI analyses, selecting the left amygdala as a seed region and observing what other regions were functionally coupled with it during the encoding conditions.

**Figure 4:**
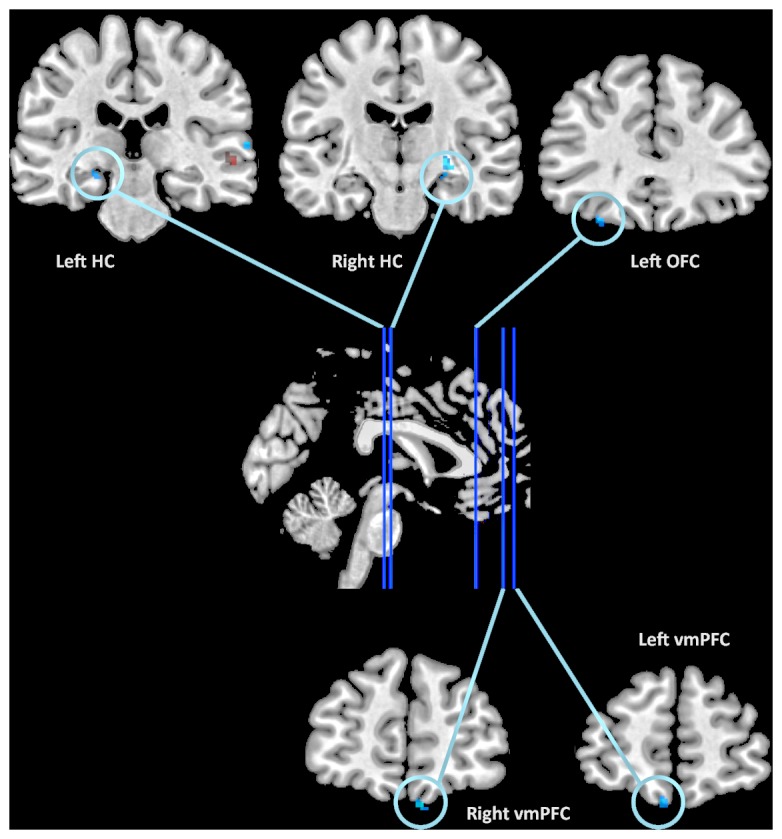
Regions that correlated significantly with left amygdala during psychophysiological interaction. Regions in blue showed a significant negative correlation with left amygdala during negative integration; regions in red showed a significant positive correlation during neutral integration.

For neutral integration greater than negative integration, we found left amygdala activity correlated with bilateral HC (24 −16 −12, *k* = 47; −20 −22 −12, *k* = 31), left orbitofrontal cortex (OFC; −40 40 −20, *k* = 60) and medial OFC (−4 56 −22, *k* = 15). For negative integration greater than neutral integration, we found left amygdala activity correlated with right PHG (32 −14 −26, *k* = 10). By using these full interaction contrasts to mask PPIs generated from the individual conditions versus baseline (e.g., negative integration vs. baseline), we were able to infer the directionality of these correlations (Fig. 4; see Methods). During neutral integration, only one region within auditory cortex reached significance, showing a positive correlation with amygdala engagement. During negative integration, left amygdala activity was negatively correlated with bilateral HC (26 −16 −10, *k* = 19; −22 −20 −12, *k* = 17), right ventromedial PFC (8 52 −26, *k* = 35), and bilateral OFC (36 42 −12, *k* = 7; −28 36 −22, *k* = 5; −6 56 −22, *k* = 9). At the *P*<0.01 threshold, no regions were positively correlated with left amygdala during negative integration. See Table 3 for all activations.

**Table 3:** Regions showing significant psychophysiological interaction with left amygdala activity.

Region	Laterality	x	y	z	t	k	Correlation
vmPFC	R	8	52	−26	4.61	35	Neg −
HC	R	26	−16	−10	3.56	19	Neg −
OFC	R	36	42	−12	3.27	7	Neg −
OFC	L	−28	36	−22	3.11	5	Neg −
OFC	L	−6	56	−22	3.06	9	Neg −
Striatum	R	26	−14	−4	3.01	19	Neg −
HC	L	−22	−20	−12	2.92	17	Neg −
Auditory Cortex	R	56	−26	2	2.83	28	Neu +
Auditory Cortex	R	66	−24	8	2.71	32	Neg −

## Discussion

The present study tested and found support for the hypothesis that the regions supporting integration would differ based on the emotional content of the integration. During both encoding and retrieval, activity in visual processing regions supported the encoding and retrieval of emotional integrations, while activity in PFC and PHG supported the encoding and retrieval of neutral integrations. These data suggest that individuals have two distinct routes to integrative success, and the likelihood of their engagement depends on the emotional content to be integrated.

Staresina and Davachi [10] suggested that integration could take place within the visual processing stream, before information even reaches MTL. Although there were several major differences between that paper and the present report (including the format and emotionality of stimuli used), our results offer support for the idea that integration can be supported by visual processing regions. However, our results also suggest that when the integrations are supported by mental imagery, as in the present study, the contribution of visual processing regions is specific to integrations that contain emotional information. Together, these results indicate that emotional and neutral integrations rely on separable neural processes for successful encoding and retrieval. Indeed, parametric analyses offer strong support for this dissociation: not only is the integration of emotional pairs selectively associated with visual activity and the integration of neutral pairs selectively associated with PFC and MTL activity, but in each case activity in those regions increases as a function of encoding success.

Analyses using PPI as a method of functional connectivity revealed that the decreased reliance on prefrontal and MTL activity for emotional integrations was likely related to amygdala engagement. These analyses revealed a negative correlation between the amygdala and the MTL and vmPFC/OFC during negative integration: Increases in left amygdala activity during negative integration were related to decreases in activity in those regions. This suggests that when participants encoded images that elicited an emotional reaction and amygdala activity, those frontal and temporal regions were less involved in the integration process. This result emphasizes that amygdala engagement may not only facilitate processing, as has often been described [19], [29]–[31] but may also be connected to disruptions in encoding processes.

The current data present a compelling case that younger adults utilize two routes to successfully creating and remembering integrated associative representations: a visual route for integrations containing emotional information and a frontal- and medial-temporal route for integrations that contain only neutral information. Moreover, successful memory retrieval is supported by similar routes to those observed at encoding: successful recognition of previously- integrated neutral pairs is supported by a region of posterior PHG, while successful recognition of previously-integrated emotional pairs is supported by cuneus. Thus, these data inform the discussion about whether special types of associations – such as integrations – are supported by perceptual (e.g., visual) processes or conceptual (e.g., frontal/temporal) processes: the neural processes that support integration depend on the emotionality of information being integrated.

There are important limitations to this research. First, our decision to assess neural processing under conditions that minimized behavioral effects of emotion brought with it some benefits but may have also made it harder to detect differences between the emotional and neutral conditions. It will be fruitful for future research to examine whether there are larger differences in the mechanisms supporting emotional and neutral integrations under conditions that exaggerate the differences in performance for those pairs. More generally, many of the effects reported here are weak, at the border of significance. Because we are looking for subtle effects – the interactive effects of changing the emotionality of a single word and of asking participants to imagine that concept interacting with another concept rather than separately from it – it is possible that our study is underpowered. The number of trials we could show each participant was limited by the length of time that participants could lie comfortably in the fMRI scanner. Future research could increase power not only by using a large sample of participants but also by choosing to scan at only encoding or retrieval (and not at both phases as was done here), enabling a larger number of trials to be presented to each participant. Second, we only assessed memory after one time-point, and therefore all of our conclusions are based on the successful retrieval of integrations after an approximately 30-minute delay. Taken together with our prior behavioral data [2] – which suggested a faster method of integration for emotional pairs than neutral ones, but a reduced maintenance of those traces in memory – it will be interesting for future research to examine whether the integrations formed using primarily visual processes, rather than additional PFC and MTL processes, may be less likely to be retained over longer delays. Third, we had relatively little variability in the self-reported visual imagery ability of our participants. It is likely that individuals with low imagery ability recruit different processes during this task than those with high imagery ability [32]–[34]. It will be important for future research to examine whether imagery ability influences the effects of emotion on integrative imagery success.

These results are an important step in understanding why events that contain emotional information are often remembered differently than events that do not contain emotional information, particularly in cases where emotional information either impedes [35]–[36] or facilitates [37]–[39] the formation of novel associations.

## Conclusions

It has been debated whether special types of associations, such as integrations, can be formed within visual processing regions [10] or whether the medial temporal lobes are required for their successful creation and retrieval [5]–[7], [9]. Here we show that the answer depends on the emotional content of the integrated information. The encoding and retrieval of emotional integrations rely disproportionately on visual processing regions, specifically cuneus and precuneus, while neutral integrations rely heavily on prefrontal and medial temporal lobe regions. Moreover, we raise the possibility that amygdala activity during the integration of negative pairs may potentially have a disruptive effect on frontal and temporal processing, with increased activity in left amygdala negatively correlating with reduced activity in bilateral vmPFC/OFC and bilateral HC.

## References

[1] Wollen KA, Weber A, Lowry DH (1972) Bizareness versus interaction of mental images as determinants of learning. Cogn Psychol 3: 518–523.

[2] Murray BD, Kensinger EA (2012) The effects of emotion and encoding strategy on associative memory. Mem Cognit 40: 1056–69. 10.3758/s13421-012-0215-3 22592895

[3] Aggleton JP, Brown MW (1999) Episodic memory, amnesia, and the hippocampal- anterior thalamic axis. Behav Brain Sci 3: 425–444. 11301518

[4] Eichenbaum H, Yonelinas AP, Ranganath C (2007) The medial temporal lobe and recognition memory. Annu Rev Neurosci 30: 123–152. 17417939 10.1146/annurev.neuro.30.051606.094328 PMC2064941

[5] Giovanello KS, Keane MM, Verfaellie M (2006) The contribution of familiarity to associative memory in amnesia. Neuropsychologia 44: 1859–1865. 16643967 10.1016/j.neuropsychologia.2006.03.004 PMC1698551

[6] Quamme JR, Yonelinas AP, Norman KA (2007) Effect of unitization on associative recognition in amnesia. Hippocampus 17: 192–200. 17203466 10.1002/hipo.20257

[7] Staresina BP, Davachi L (2006) Differential encoding mechanisms for subsequent associative recognition and free recall. J Neurosci 26: 9162–9172. 16957073 10.1523/JNEUROSCI.2877-06.2006 PMC6674493

[8] Haskins AL, Yonelinas AP, Quamme JR, Ranganath C (2008) Perirhinal cortex supports encoding and familiarity-based recognition of novel associations. Neuron 59: 554–60. 18760692 10.1016/j.neuron.2008.07.035

[9] Staresina BP, Davachi L (2008) Selective and shared contributions of the hippocampus and perirhinal cortex to episodic item and associative encoding. J Cogn Neurosci 21: 1478–1489. 10.1162/jocn.2008.20104 PMC2789239 18303974

[10] Staresina BP, Davachi L (2010) Object unitization and associative memory formation are supported by distinct brain regions. J Neurosci 30: 9890–9897. 20660271 10.1523/JNEUROSCI.0826-10.2010 PMC2927970

[11] Vuilleumier P, Armony JL, Driver J, Dolan RJ (2001) Effects of attention and emotion on face processing in the human brain: An event-related fmri study. Neuron 30: 829–841. 11430815 10.1016/s0896-6273(01)00328-2

[12] Compton RJ (2003) The interface between emotion and attention: A review of evidence from psychology and neuroscience. Behav Cogn Neurosci Rev 2: 115–129. 13678519 10.1177/1534582303255278

[13] Vuilleumier P, Richardson MP, Armony JL, Driver J, Dolan RJ (2004) Distant influences of amygdala lesion on visual cortical activation during emotional face processing. Nat Neurosci 7: 1271–1278. 15494727 10.1038/nn1341

[14] Mather M, Mitchell KJ, Raye CL, Novak DL, Greene EJ, et al (2006) Emotional arousal can impair feature binding in working memory. J Cogn Neurosci 18: 614–625. 16768364 10.1162/jocn.2006.18.4.614

[15] Richardson MP, Strange BA, Dolan RJ (2004) Encoding of emotional memories depends on amygdala and hippocampus and their interactions. Nat Neurosci 7: 278–285. 14758364 10.1038/nn1190

[16] Phelps EA (2004) Human emotion and memory: Interactions of the amygdala and hippocampal complex. Curr Opin Neurobiol 14: 198–202. 15082325 10.1016/j.conb.2004.03.015

[17] McGaugh JL, McIntyre CK, Power AE (2002) Amygdala modulation of memory consolidation: Interaction with other brain systems. Neurobiol Learn Mem 78: 539–552. 12559833 10.1006/nlme.2002.4082

[18] Dolcos F, LaBar K, Cabeza R (2004) Interaction between the amygdala and the medial temporal lobe memory system predicts better memory for emotional events. Neuron 42: 855–863. 15182723 10.1016/s0896-6273(04)00289-2

[19] Anderson AK, Phelps EA (2001) Lesions of the human amygdala impair enhanced perception of emotionally salient events. Nature 411: 305–309. 11357132 10.1038/35077083

[20] Libkuman TM, Stabler CL, Otani H (2004) Arousal, valence, and memory for detail. Memory 12: 237–247. 15250188 10.1080/09658210244000630

[21] Graf P, Schacter DL (1989) Unitization and grouping mediate dissociations in memory for new associations. J Exp Psychol Learn Mem Cog 15: 930–940. 10.1037//0278-7393.15.1.3 2522139

[22] Bradley MM, Lang PJ (1999) Affective norms for English words (ANEW): Stimuli, instruction manual and affective ratings. Technical report C-1, Gainesville, FL. The Center for Research in Psychophysiology, University of Florida.

[23] Kuèera M, Francis W (1967) Computational analysis of present-day American English. Providence, RI: Brown University Press.

[24] Stark CE, Squire LR (2001) When zero is not zero: The problem of ambiguous baseline conditions in fMRI. Proc Natl Acad Sci U S A 98: 12760–12766. 11592989 10.1073/pnas.221462998 PMC60127

[25] Kirwan CB, Stark CE (2004) Medial temporal lobe activation during encoding and retrieval of novel face-name pairs. Hippocampus 14: 919–930. 15382260 10.1002/hipo.20014 PMC2704554

[26] Nielsen FA, Hansen LK (2002) Automatic anatomical labeling of Talairach coordinates and generation of volumes of interest via the BrainMap database. NeuroImage 2 presented at the 8th annual conference on functional mapping of the human brain. Available: http://hendrix.imm.dtu.dk/services/jerne/ninf/voi.html.

[27] Fox PT, Mikiten S, Davis G, Lancaster JL (1994) BrainMap: A database of human function brain mapping. In Thatcher RW, Hallet M, Zeffiro T, John ER, & Huerta M, editors. Functional neuroimaging: Technical foundations.San Diego: Academic press. pp. 95–105.

[28] Slotnick SD (2008) Cluster threshold beta. Available: http://www2.bc.edu/~slotnics/scripts.htm.

[29] Cahill L, McGaugh JL (1995) A novel demonstration of enhanced memory associated with emotional arousal. Conscious Cogn 4: 410–421. 8750416 10.1006/ccog.1995.1048

[30] Hamann S (2001) Cognitive and neural mechanisms of emotional memory. Trends Cogn Sci 5: 394–400. 11520704 10.1016/s1364-6613(00)01707-1

[31] Öhman A, Flykt A, Esteves F (2001) Emotion drives attention: detecting the snake in the grass. J Exp Psychol Gen 130: 466–478. 11561921 10.1037/0096-3445.130.3.466

[32] Marks DF (1995) New directions for mental imagery research. Journal of Mental Imagery 19: 153–167.

[33] Blajenkova O, Kozhevnikov M, Motes MA (2006) Object-Spatial Imagery: A new self-report imagery questionnaire. Appl Cogn Psychol 20: 239–263.

[34] Campos A, Pérez-Fabello MJ (2009) Psychometric quality of a revised version Vividness of Visual Imagery Questionnaire. Percept Mot Skills 108: 798–802. 19725316 10.2466/PMS.108.3.798-802

[35] Jacobs WJ, Nadel L (1998) Neurobiology of reconstructed memory. Psychol Public Policy Law 4: 1110–1134.

[36] Payne JD, Nadel L, Britton WB, Jacobs WJ (2004) The biopsychology of trauma and memory. In Reisberg D & Hertel P, editors. Memory and emotion. London: Oxford. pp 76–128. University Press.

[37] MacKay DG, Ahmetzanov MV (2005) Emotion, memory, and attention in the taboo Stroop paradigm: An experimental analogue of flashbulb memories. Psychol Sci 16: 25–32. 15660848 10.1111/j.0956-7976.2005.00776.x

[38] MacKay DG, Shafto M, Taylor JK, Marian DE, Abrams L, et al (2004) Relations between emotion, memory, and attention: Evidence from taboo Stroop, lexical decision, and immediate memory tasks. Mem Cognit 32: 474–488. 10.3758/bf03195840 15285130

[39] Hadley CB, MacKay DG (2006) Does emotion help or hinder immediate memory? Arousal versus priority-binding mechanisms. J Exp Psychol Learn Mem Cog 32: 79–88. 10.1037/0278-7393.32.1.79 16478342

